# Effects of dietary supplementation of organic acids and phytase on performance and intestinal histomorphology of broilers

**Published:** 2016-09-15

**Authors:** Nasibeh Mohammadagheri, Ramin Najafi, Gholamreza Najafi

**Affiliations:** 1 *Department of Animal Science, Faculty of Agriculture, Urmia University, Urmia, Iran; *; 2 *Department of Basic Science, Faculty of Veterinary Medicine, Urmia University, Urmia, Iran.*

**Keywords:** Broiler chicken, Intestinal histomorphology, Organic acids, Phytase enzyme

## Abstract

The present experiment was conducted to evaluate the effects of organic acids and phytase enzyme supplementation on performance and intestinal histomorphology of broilers. The experiment was done in a factorial arrangement 2 × 2 × 2 based on completely randomized design with eight treatments, five replicates with 12 chicks in each until 42 days of age. Diets included natural vinegar (0 and 2%), citric acid (CA; 0.00 and 1.00%) and phytase enzyme (PHY; 0.00 and 500 FTU phytase per kg of feed). One bird from each treatment replicate was randomly selected and slaughtered to evaluate the small intestinal morphology on 42 days of age. Analysis of results showed that vinegar increased feed consumption and body weight gain in total experimental period (*p *˂ 0.05), while CA significantly decreased feed consumption on 0-14 days of age (*p *˂ 0.05). No effect was observed on performance in interaction of organic acids together and with PHY group (*p *> 0.05). In duodenum CA increased the villus height and width (*p *˂ 0.05) and PHY enzyme increased villus width (*p *˂ 0.05) and decreased crypt depth (*p *˂ 0.05). On the other hand, CA along with PHY significantly decreased crypt depth (*p *˂ 0.05). In jejunum PHY alone and in combination with vinegar increased the goblet cells numbers (*p *˂ 0.05), whereas vinegar significantly increased the goblet cells numbers in ileum (*p *˂ 0.05). The muscular thickness in duodenum, jejunum, and ileum was not aﬀected among diﬀerent treatment groups. The results showed that supplementation of organic acids and phytase together in this experiment, with no negative effects on each other, improved their effects on some parameters.

## Introduction

Antibiotic growth promoters have long been added as feed supplements for livestock in order to improve performance as well as for the therapeutic and pro-phylactic purposes.^1^ They stabilize the intestinal microbial flora and lead to improve performance and reduce the pathogen load and mortality.^2^ The antibiotic resistant genes can be transmitted from animal to human microbiota.^3^ Efforts have been made to find replacements for antibiotics that have a positive effect on the growth performance of broilers.^4^ Several alternatives for antibiotic growth promoters have been proposed such as organic acids, probiotics, phytogenic feed additives and products of enzymes.^5^

For decades, organic acids have been used in feed preservation, either for protecting feed from microbial or fungal destruction. Organic acids are widespread in plant and animal tissues. It was reported that propionic, formic, citric, lactic and ascorbic acids increase nutrient digestibility without harmful affecting performance.^6^ Organic acids can improve animal performance due to their antimicrobial activity, which improve protein and energy digestibility thereby reducing microbial competition with the host for nutrients and endogenous nitrogen losses, as well as by lowering the incidence of subclinical infections and the secretion of immune mediators.^7^ Apart from the antimicrobial activity, they reduce the pH of digesta, increase the pancreatic secretion and have trophic effects on the mucosa of gastrointestinal tract.^8^ Health of the gut is one of the major factors governing the performance of birds and thus, the profile of intestinal microflora plays an important role in gut health.^9^

The intestinal microflora of broilers have beneficial effects on the nutrients, making them more available from the intestinal tract by affecting apparent digestibility of protein, nitrogen, and fat, depending on the composition of the diet.^4^ Organic acids inhibit the growth of intestinal bacteria leading to reduce metabolic needs by increasing the availability of nutrients to the host.^10^ In most cereal grains and oilseeds the phytate phosphorus (PP) is the main storage form of phosphorus and represents 50.00 to 85.00% of the total phosphorus.^11^ Phytate phosphorus is not available to the poultry because they do not contain sufficient amounts of intrinsic phytase (PHY) required to hydrolyze the PP.^12^ Since there is no natural PHY in the gastrointestinal tract of the poultry, supplementation of diets with exogenous PHY has been proven to enhance the digestibility of phytate phosphorus.^13^ Citric acid (CA) can improve the efficacy of PHY because it can chelate multivalent cations.^14^ Acidiﬁcation with various organic acids has been reported to reduce the production of toxic components by the bacteria and colonization of pathogens on the intestinal wall, thus preventing damage to epithelial cells.^15^

There is limited information about the effects of organic acids and PHY supplementation on the performance and intestinal histomorphology of the broiler chickens and the objectives of this study was to evaluate them.

## Materials and Methods

Birds and dietary treatments. One-day-old male Ross 308 broiler chickens (n = 480) were individually weighed and pen averages were adjusted so that no significant difference among pens was realized. The chickens were allocated to 40 pens in a 2 × 2 × 2 factorial arrangement based on completely randomized design with eight treatments, five replicates and 12 chickens in each. Wood shavings were used as litter. A basal diet was formulated with wheat-corn-soybean meal according to the Ross 308 recommendations for all nutrients.^16^ Treatments included: 1) basal diet, 2) basal diet + 2.00% natural grape vinegar, 3) basal diet + 1.00% citric acid, 4) basal diet + 500 FTU kg^-1^ PHY, 5) basal diet + 2.00% natural grape vinegar + 1.00% CA, 6) basal diet + 2.00% natural grape vinegar + 500 FTU kg^-1^ PHY, 7) basal diet + 1.00% citric acid + 500 FTU kg^-1^ PHY, 8) basal diet + 2.00% natural grape vinegar + 1.00% CA + 500 FTU kg^-1^ PHY. Ingredient composition and the calculated nutrients composition are given in Table 1. The temperature was regulated at 32 ± 1 ˚C in the first week and reduced by 2 ˚C per week to receive 21 ˚C in the sixth week. Feed and water were provided ad libitum and a continuous lighting schedule were used throughout the experimental period.

Measurements. The body weight gain of birds per replicate was recorded on the individual basis at weekly intervals. The cumulative feed consumption per replicate was also recorded on a weekly basis. Feed conversion ratio per replicate was worked out at weekly intervals by taking into consideration the weekly body weight gain and the feed consumption of respective replicate. For histomorphological assessment, at the end of the experiment (42^nd^ day of age), five broiler chickens of similar body weight with the group average were selected from each group and slaughtered by severing a jugular vein. Samples of duodenum, jejunum and ileum were obtained from the slaughtered birds and approximately two cm of the middle portions of the duodenum, jejunum and ileum were excised and fixed in 10% buffered formalin for one week. Tissues were dehydrated by immersing through a series of alcohols with increasing concentration (from 70% to absolute), infiltrated with xylene and embedded in paraffin.

The rotary type microtome was used for cutting the paraffin sections (7.00 µm). The blocks were properly trimmed and the sections of 7.00 µm thickness were cut. The tissue sections were stained by hematoxylin and eosin for measuring the villus height, villus width, crypt depth and muscular thickness. Acidic mucus containing goblet cells were identified using periodic acid shift (PAS). All of the specimens were studied by multiple magnifications (400× and 1000×).^17^ The experimental protocols were reviewed and approved by the Animal Care Committee of the Urmia University, Urmia, Iran.

Statistical analysis. Data were analyzed using the general linear models (GLM) procedure in SAS software (Version 9.12; SAS Institute, Cary, USA) with the main effects of natural grape vinegar, CA, and PHY and their two- and three-way interactions included in the model. The differences among group means were verified statistically by analysis of variance using Tukey’s test at p ˂ 0.05. The following model statement was used:^4^

 Y_ijkl_ = μ + P_i_ + C_j_ + S_k_ + PC_ (ij)_ + PS_ (ik)_ + CS_ (jk)_ + PCS_ (ijk)_ + e_ijkl_

where, Y_ijkl_ is the response measured, μ is the overall mean, P_i_ is the effect of PHY, C_j_ is the effect of natural grape vinegar level, S_k_ is the effect of CA level, PC_(ij)_ is the interaction between PHY and natural grape vinegar, PS_(ik)_ is the interaction between PHY and CA, CS_(jk)_ is the interaction between natural grape vinegar and CA, PCS_(ijk)_ is the interaction among natural grape vinegar, CA acid, and PHY, and e_ijkl_ is the residual error.

**Table 1:** Composition of experimental diets in g kg^-1^.

**Ingredients**	**Starter (0 to 10** ^th^ ** day)**	**Grower (11 to 24** ^th^ ** day)**	**Finisher ( 25 to 42** ^nd^ ** day)**
**Maize**	41.90	42.49	40.00
**Wheat**	14.06	20.00	25.00
**Soya bean meal**	37.50	31.20	28.15
**Soya bean oil**	2.20	2.50	3.20
**Limestone**	1.00	0.80	0.80
**Di-calcium phosphate**	2.20	2.00	1.80
**DL-methionine**	0.18	0.15	0.15
**L- lysine**	0.16	0.06	0.10
**Vitamin premix** ∗	0.25	0.25	0.25
**Trace mineral mixture** ∗∗	0.25	0.25	0.25
**Salt**	0.30	0.30	0.30
***Analyzed Values***			
**Metabolizable energy (kcal kg** ^-1^ ** diet)**	2860	2950	3020
**Crude protein (%)**	21.23	19.05	18.00
**Calcium (%)**	0.96	0.84	0.79
**Available phosphorus (%)**	0.47	0.43	0.39
**Methionine %**	0.49	0.44	0.42
**Methionine + Cystine (%)**	0.86	0.78	0.75
**Lysine (%)**	1.28	1.06	1.02

∗ Vitamin premix (per 2.5 kg); Vitamin A: 10,000,000 IU, Vitamin D_3_: 5000,000 IU, Vitamin E: 50,000 IU, Vitamin K_3_: 2 g, Vitamin B_1_: 2 g, Vitamin B_2_: 6 g, Niacin: 40 g, Vitamin B_6_: 4 g, Vitamin B_12_: 16 mg, Folic acid: 1.75 g, D-biotin: 150 mg, Ca-D-pantothenate: 13 g, Carophyll-yellow: 25 g, and Antioxidant: 12.5 g.

∗∗ Trace mineral premix (per 2.5 kg); Mn: 120 g, Fe: 40 g, Zn: 100 g, Cu: 16 g, I: 1 g, Se: 0.2 g, and choline chloride: 400 g.

## Results

**Performance. **Effect of dietary treatments on growth performance, including body weight gain (BWG), feed intake (FI) and feed conversion ratio (FCR), are summarized in Table 2. Dietary supplementation of vinegar signiﬁcantly increased FI and the BWG in total experimental period (*p *˂ 0.05) whereas there was not any notable effect on 0 to 14 days of age. The CA and PHY did not have any effect on BWG. The interaction of organic acids together and with PHY did not have any effect on performance.

**Histomorphology. **The mean values regarding the histomorphological alterations in broilers fed organic acids and PHY are given in Tables 3 to 5. The analysis of results showed that CA increased the villusheight and width in duodenum (*p *˂ 0.05). The PHY increased the villuswidth (*p *˂ 0.05) and decreased the crypt depth (*p* ˂ 0.05) and in combination with CA decreased the crypt depth and increased villus height to crypt depth ratio significantly (*p *˂ 0.05) while vinegar alone or in combination with PHY did not have any significant effect on characteristics of duodenum. In jejunum, PHY alone and in combination with vinegar increased the goblet cell numbers (*p *˂ 0.05) whereas vinegar significantly increased the goblet cell numbers in ileum (*p *˂ 0.05). The muscular thickness in the duodenum, jejunum and ileum was not aﬀected by diﬀerent treatment groups. Citric acid and PHY alone and together increased the ratio of villus height to crypt depth in duodenum (*p *˂ 0.05).

**Table 2:** Effects of organic acids and phytase supplementation on the performance of broilers in different days

**Treatments**		**Feed consumption (g)**			**weight gain (g)**			**Feed conversion ratio**		
		**(0-14)**	**(15-28)**	**(29-42)**	**(0-14)**	**(15-28)**	**(29-42)**	**(0-14)**	**(15-28)**	**(29-42)**
**Vinegar (%)**	**0.00**	440^b^	1291^b^	2234^b^	350	840^b^	1163^b^	1.25	1.53	1.92
	**2.00**	460^a^	1384^a^	2418^a^	361	888^a^	1282 a	1.27	1.55	1.88
**Citric acid (%)**	**0.00**	462^a^	1351	2381	360	879	1237	1.28	1.53	1.92
	**2.00**	438^b^	1324	2274	351	849	1209	1.24	1.55	1.88
**Phytase (FTU)**	**0.00**	454	1347	2362	352	877	1248	1.28	1.53	1.89
	**500**	446	1328	2290	359	851	1198	1.23	1.56	1.91
**Standard Error**		6.00	19.00	39.00	4.70	15.00	40.00	0.01	0.02	0.03
***p-value***										
**Vinegar**		0.01	0.001	002	0.07	0.03	0.01	0.27	0.34	0.45
**Citric acid**		0.01	0.57	0.51	0.62	0.45	0.35	0.14	0.18	0.19
**Phytase**		0.32	0.57	0.68	0.74	0.75	0.67	0.18	0.42	0.12
**Vinegar × Citric **		0.38	0.87	0.64	0.56	0.08	0.39	0.60	0.46	0.34
**Vinegar × Phytase**		0.12	0.06	0.96	0.05	0.75	0.23	0.19	0.19	0.20
**Citric × Phytase**		0.08	0.64	0.35	0.25	0.65	0.45	0.15	0.79	0.17
**Vinegar × Citric × Phytase**		0.12	0.34	0.40	0.57	0.31	0.51	0.45	0.23	0.92

ab Different superscript letters within a column indicate significant differences at *p *˂ 0 .05.

**Table 3:** Effects of organic acids and phytase supplementation on histomorphology of duodenum of broilers at 42^nd ^day

**Treatments**		**Villus ** **height** **(µm)**	**Villus width (** **µm)**	**Crypt depth ** **(µm)**	**Epithelium thickness ** **(µm)**	**Number of Goblet cells**	**Villus ** **height** **/** **Crypt depth ratio**
**Vinegar (%)**	**0.00**	1361	139	143	199	867	9.51
	**2.00**	1346	140	142	209	876	9.47
**Citric acid (%)**	**0.00**	1337^b^	136^b^	145	199	868	9.22^b^
	**2.00**	1370^a^	141^a^	141	201	876	9.71^a^
**Phytase (FTU)**	**0.00**	1356	137^b^	146^a^	199	870	9.28^b^
	**500**	1351	141^a^	140^b^	201	875	9.65^a^
**Standard Error**		10.00	1.10	1.30	2.20	8.00	0.12
***p-value***							
**Vinegar**		0.31	0.38	0.57	0.72	0.51	0.54
**Citric acid**		0.04	0.02	0.07	0.57	0.51	0.01
**Phytase**		0.74	0.03	0.03	0.57	0.68	0.02
**Vinegar × Citric **		0.56	0.08	0.39	0.87	0.64	0.90
**Vinegar × Phytase**		0.38	0.75	0.08	0.06	0.96	0.06
**Citric × Phytase**		0.25	0.08	0.04	0.64	0.07	0.00
**Vinegar × Citric × Phytase**		0.06	0.31	0.51	0.08	0.40	0.06

ab Different superscript letters within a column indicate significant differences at *p *˂ 0 .05.

**Table 4:** Effects of organic acids and phytase supplementation on histomorphology of jejunum of broilers at 42^nd ^day

**Treatments**		**Villus ** **height** **(µm)**	**Villus width (** **µm)**	**Crypt depth ** **(µm)**	**Epithelium thickness ** **(µm)**	**Number of Goblet cells**	**Villus ** **height** **/** **Crypt depth ratio**
**Vinegar (%)**	**0.00**	1196	150	160	217	1038	7.47
	**2.00**	1147	149	157	214	1059	7.30
**Citric acid (%)**	**0.00**	1170	146	161	218	1057	7.26
	**2.00**	1173	152	156	213	1040	7.52
**Phytase (FTU)**	**0.00**	1155	147	159	212	1021^b^	7.26
	**500**	1188	150	157	218	1076^a^	7.57
**Standard Error**		23.00	2.00	2.00	2.00	11.00	0.16
***p-value***							
**Vinegar**		0.16	0.77	0.34	0.45	0.32	0.47
**Citric acid**		0.94	0.12	0.12	0.19	0.44	0.32
**Phytase**		0.35	0.35	0.42	0.07	0.01	0.18
**Vinegar × Citric **		0.28	0.39	0.46	0.03	0.48	0.54
**Vinegar × Phytase**		0.14	0.45	0.19	0.20	0.04	0.05
**Citric × Phytase**		0.36	0.70	0.79	0.17	0.22	0.32
**Vinegar × Citric × Phytase**		0.31	0.45	0.23	0.92	0.66	0.12

ab Different superscript letters within a column indicate significant differences at *p *˂ 0 .05.

**Table 5:** Effects of organic acids and phytase supplementation on histomorphology of ileum of broiler chicken at 42^nd^ day

**Treatments**		**Villus ** **height** **(µm)**	**Villus width (** **µm)**	**Crypt depth ** **(µm)**	**Epithelium thickness ** **(µm)**	**Number of Goblet cells**	**Villus ** **height** **/** **Crypt depth ratio**
**Vinegar (%)**	**0.00**	892	154	136	232	1194^b^	6.55
	**2.00**	908	158	142	231	1264^a^	6.39
**Citric acid (%)**	**0.00**	914	156	140	229	1226	6.52
	**2.00**	886	157	138	234	1231	6.42
**Phytase (FTU)**	**0.00**	908	157	141	234	1208	6.43
	**500**	892	155	138	230	1250	6.46
**Standard Error**		9.00	2.60	2.60	2.00	19.00	0.13
***p-value***							
**Vinegar**		0.26	0.30	0.13	0.84	0.01	0.36
**Citric acid**		0.06	0.84	0.56	0.10	0.85	0.57
**Phytase**		0.25	0.55	0.43	0.23	0.13	0.93
**Vinegar × Citric **		0.04	0.34	0.34	0.00	0.42	0.04
**Vinegar × Phytase**		0.70	0.04	0.06	0.43	0.28	0.05
**Citric × Phytase**		0.31	0.94	0.62	0.76	0.36	0.95
**Vinegar × Citric × Phytase**		0.19	0.07	0.07	0.07	0.38	0.05

ab Different superscript letters within a column indicate significant differences at *p *˂ 0 .05.

## Discussion

 Kishi et al. reported that improved body weight gain is probably due to the beneﬁcial effects of organic acids on the gut ﬂora.^18^ The organic acids may aﬀect the integrity of microbial cell membrane or cell macromolecules or interfere with the nutrient transport and energy metabolism causing the bactericidal effect. In this study, the possible improvement in performance by vinegar may be attributed to the nutrients in vinegar. Acetic acid is the main component of vinegar. Some other constituents include, anthocyanins (e.g. cyanidin-3- glucoside) flavonols (e.g. quercetin, kaempferol), flavanols (catechin, epicatechin),^19^ vitamins, mineral salts, amino acids and nonvolatile organic acids (e. g. tartaric, citric, malic, lactic).^20^ Therefore, vinegar has more effects rather than citric acid on performance of broilers. Addition of vinegar to mash diet may lead to increase in palatability and improve mixing ration items, resulting in increased FI and consequently an increase in BWG. These results were in concordance with the reports of earlier researchers. Abdel-Fattah et al. ^21^ reported that the addition of dietary acetic acid improved live body weight of broiler chicks compared to those fed on unsupplemented diets. Kopecký et al. concluded that supplementation of citric acid caused decrease in total feed consumption;^5^ that was in agreement with the results of Afsharmanesh and Pourreza, who reported that the addition of citric acid to broiler diet improved feed efficiency. ^22^ Sacakli et al. found that feed consumption and feed conversion ratio were not affected by addition of PHY or organic acid alone or in combination to diet.^6^ In the same way, Houshmand et al.,^23^ and Hernandez et al., have also observed no significant effect of organic acid supple-mentation on growth performance of broilers.^24^ Adil et al. reported that supplementation of organic acids in broilers improved BWG when compared to the unsupplemented group.^10^ They reported that improved BWG is probably due to the beneﬁcial effects of organic acids on the gut ﬂora.^10^ Numerous factors, such as environment, farm rearing conditions, management practices, nutrition, organic acid type and concentration, composition of diet and bird characteristics (age, species and stage of production) can affect the response of broilers to feed additives.^8^^,^^25^ Thus, effects of citric acid in the present study could be attributed to above mentioned factors. Results on growth-promoting effects of organic acids have been inconsistent.^26^ This inconsistency in results could be attributed to several factors. One of the most important factors is the buffering capacity of dietary feedstuffs. The beneficial effects of antimicrobial additives on growth performance would be apparent under poor hygienic conditions or when feeding poorly digestible diets.^26^ This study was carried out under good rearing conditions. Under good conditions, broilers did not need any feed additives for maximum growth performance.^27^

Height and width of villus could be considered as indicators for an active functioning of intestine.^28^ Increased villus height provides a greater surface area for nutrients absorption and consequently, higher performance.^28^ On the contrary, reduction in villus height can reduce nutrients absorption due to the decrease in the intestinal surface area for absorption. Thus, reducing nutrients absorption decreases resistance to disease and lower growth performance. Increase in secretions of gastrointestinal tract are the negative consequence of deeper crypt and shorter villi.^29^ Pelicano et al. reported increased villus heights in duodenum and jejunum with most of the organic acidiﬁers which they attributed to the fact that organic acids reduce the growth of many pathogenic or nonpathogenic intestinal bacteria, decreasing the intestinal colonization and infectious processes, ultimately decreasing the inﬂammatory reactions at the intestinal mucosa, which increases the villus height and functions of secretion, digestion and absorption of nutrients by the mucosa.^30^ The reduction in the muscular thickness is helpful in improving the digestion and absorption of nutrients as reported by Teirlynck et al.^31^ The thickening of mucous layer on the intestinal mucosa contributes to the reduced digestive eﬃciency and nutrients absorption.^31^ Garcia et al. reported that broiler chickens fed on formic acid had the greater villus height and width, and crypt depth compared to the control group.^26^ Reportedly, reported that the duodenum villus height was significantly increased with the different levels of CA compared to the control.^21^^,^^32^ Moreover, the presence of other antimicrobial compounds, organic acid type and concentration, composition of diet and the experimental environment are factors that could affect response of birds to organic acids.^8^

In conclusion, this study pointed out the importance of using organic acid as physiological additives to increase the growth performance and intestinal histomorphology of broilers through their physiological action in inducing the growth and activities of some endogenous mechanisms responsible for better performance. As well, under the condition of this study, no further benefits were achieved as a result of increasing the dietary organic acids and PHY levels. The reason of these results may be due to type and dose of organic acids and PHY in these studies. Further studies are needed to throw more light on developmental effects of those organic acids on the performance and intestinal histomorphology of broiler chickens.
